# Nursing documentation practice and associated factors among nurses in public hospitals, Tigray, Ethiopia

**DOI:** 10.1186/s13104-019-4661-x

**Published:** 2019-09-23

**Authors:** Hagos Tasew, Teklewoini Mariye, Girmay Teklay

**Affiliations:** Department Nursing, College of Health Science, Aksum University, Aksum, Ethiopia

**Keywords:** Practice, Documentation, Nurses, Associated factors

## Abstract

**Objective:**

The objective of this study was to investigate documentation practice and factors affecting documentation practice among nurses working in public hospital of Tigray region, Ethiopia.

**Results:**

In this study, there were 317 participants with 99.7% response rate. The result of this study shows that practice nursing care documentation was inadequate (47.8%). Inadequacy of documenting sheets AOR = 3.271, 95% CI (1.125, 23.704), inadequacy of time AOR = 2.205, 95% CI (1.101, 3.413) and with operational standard of nursing documentation AOR = 2.015, 95% CI (1.205, 3.70) were significantly associated with practice of nursing care documentation. To conclude, more than half of nurses were not documented their nursing care. Employing institutions should provide training on documentation of nursing care to enhance knowledge and create awareness on nurses’ documentation to nursing directors and chief executive officer to access adequate documenting supplies besides employing more nurses.

## Introduction

Nursing documentation is the record of nursing care that is planned and delivered to individual patients by qualified nurses or other caregivers under the direction of a qualified nurse [1]. Nursing documentation is the principal clinical information source to meet legal and professional requirements [2]. It is a vital component of safe, ethical and effective nursing practice whether done manually or electronically [3]. Nursing documentation should fulfill the legal requirements of nursing care documentation [4].

According to a survey done by WHO it has been shown that poor communication between health care professionals is one factor for medical errors [5]. There are also evidence indicating that nursing documentation has relationship with patient mortality [6]. Although keeping a patient record is part of their professional obligation, many studies identified deficiencies in practice of documentation among nurses across the globe [7, 8]. It has been reported that nursing records are often incomplete [8, 9], lacked accuracy and had poor quality [10, 11]. The challenges for documentation reported so far, include shortage of staff [12, 13], inadequate knowledge concerning the importance of documentation [12–15], patient load [12, 14], lack of in-service training [14, 15] and lack of support from nursing leadership [12].

As a remedy for these, many researchers recommended to use a multidisciplinary approach like to develop policies and guidelines on nursing care documentation [12, 13, 15] and provide sustained continuing training opportunities for nurses on effectiveness of documentation [7, 12, 13, 16, 17]. The nursing leaders are also expected to support, motivate [12, 17] and increase the number of staffs [15] for a better documentation practice.

Studies from South Africa and Ugandan reported deficiency in attitudes, knowledge and practice behaviors [17, 18]. The studies done in Kenya and Ghana also evidenced lack of standardized method and insufficient information of nursing documentation [12, 13]. In Ethiopia, inadequacy of data collection with lack of quality was found to be a problem [18–21]. The objective of the study was to assess nursing documentation practice and associated factors of nursing documentation practice in public hospitals of Tigray, Ethiopia.

## Main text

### Methods

A quantitative descriptive cross-sectional study design was used. The study was conducted from November 1–17, 2017. The source population for this study were all nurses who are working in government owned hospitals of Tigray region. Sample size was determined formula taking the proportion as 37.4% from previous study conducted in Northern Amhara region public hospital [14], 95% confidence interval (CI), and 5% margin of error. The final sample size was 317. Selection of hospitals for the study was carried out using simple random sampling after all hospitals in the region was identified. The study participants were selected based on the lottery method and the numbers of samples in each hospital were selected according to proportional allocation formula.

Nurses working in inpatient wards and outpatient departments; nurses having work status as a professional nurse at least for 6 months and those who were voluntary to participate were included in the study.

A structured self-administered questionnaire was developed to collect data regarding nursing documentation practice and its associated factors. Practice and knowledge of nursing documentation questions were developed based on the national guideline prepared by the FMOH (EHRIG), various books written on nursing documentation and literatures related to the topic [14, 15, 22, 23].

Prior to the actual data collection, the items were pre-tested with 5% (16 samples) of the total sample size of nurses working in Adwa hospital with self-administer questionnaire and the results were used to check reliability, consistency and completeness of the questionnaire and some improvements were done on the wordings. Reliability of the questioner was checked using Cronbach alpha (0.79).

#### Documentation practice

Practice of study participants measured using 10 multiple-choice items. A value of 3, 2, 1 and zero was scored for “always”, “sometimes”, “rarely” and “never” options respectively. For questions in which there were multiple correct and incorrect responses (n = 8), the scoring system used the proportion of correct responses [15].

#### Knowledge of documentation

The knowledge of study participants measured using 10 items with multiple options and scoring based on a number of responses given in each question. A value of 1 and 0 was scored for “yes” and “no and I don’t know” options respectively.

#### The attitude of practice

Attitude of the study participants measured by using the Likert scale questions with 10 items.

The collected data were checked for completion and cleaned manually then the data were entered into computer by SPSS version 22 software was used both for data entry and for analysis. Descriptive statistics like mean, frequency and percentage. Binary logistic regression was used for inferential statistics. Bi-variable and multivariable logistic regression were applied to measure strength of association.

#### Good practice

Those respondents who scored above or equal to the mean score of practice questions.

#### Good knowledge

Those respondents who scored above or equal to the mean score of knowledge questions [14].

#### Favorable attitude

Those respondents who scored above or equal to the mean score of attitude questions [14].

### Results

#### Socio-demographic characteristics of respondents

A 317 respondents participated in this study out of which 316 returned the questionnaires made the response rate 99.7%. From 316 nurses who participated in this study, 207 (65.5%) were females and 109 (34.5%) were males. Two hundred eight (65.5%) fall within the ranges of 25–34 years age group. Most of the respondents were holding bachelor degree 279 (88.3%). One hundred two (48.1%) of them were senior nurse professionals while 148 (46.8%) were junior nurse professionals and 11 (5.1%) were junior clinical nurses. One third of the participants were worked as a nurse for 2–5 years when 107 (33.9%) and 100 (31.6%) of them worked for more than 5 years and less than 2 years respectively **(**Table 1).

**Table 1:** Socio demographic characteristics of respondents in selected public hospitals of Tigray, Ethiopia, 2017

Variable	Frequency (n = 316)	Percent
Age group of respondents (in a years)		
< 24	50	15.8
25–34	208	65.8
35–44	33	10.4
45–54	18	5.7
55–60	7	2.2
Gender of respondents		
Male	109	34.5
Female	207	65.5
Educational level of respondents		
College diploma	18	5.7
Bachelor degree	279	88.3
MSc	19	6
Professional level of respondents		
Junior clinical nurse	16	5.1
Junior nurse professional	148	46.8
Senior nurse professional	152	48.1
Respondents’ work experience (in years)		
< 2	100	31.6
2–5	109	34.5
> 5	107	33.9
Work setting of respondents in their hospitals		
In-patient admission ward	161	50.9
Out-patient department	155	49.1

#### Practice of nurses towards nursing documentation practice

A total of 10 multiple option questions were used that had a potential score of 12 and the mean score was 7.26 (S.D ± 2.03). For this study, participant performance was categorized into good and poor practice with scores 7.26 (mean value) or above as good, while those below the mean score as having poor practice. One hundred fifty-one (47.8%) of the respondents scored to have good practice and the rest 165 (52.2%) of the study subjects scored below the mean.

Among all nurses, 230 (72.8%) of them check nursing notes written by their colleagues from which most 130 (56.5%) said the notes are incomplete. Concerning the system of documentation, majority 262 (82.2%) of them denied for application of computerized nursing documentation in their hospital. Regarding the practice of patient care documentation, most 165 (52.2%) of the respondents had poor nursing documentation practice (Table 2).

**Table 2:** Practice of nursing documentation among nurses working in selected public hospitals of Tigray, Ethiopia, 2017

Variable	Frequency (n = 316)	Percent
Nursing documentation for every patient (n = 316)		
Always	188	59.5
Sometimes	118	37.3
Rarely	8	2.5
Never	2	0.6
Time preference to document a care (n = 316)		
Any time when convenient	118	37.3
Immediately or soon after care rendered	160	50.6
At the end of shift hours	36	11.4
I don’t know	2	0.6
Ways to keep confidentiality of record (n = 316)		
Access for authorized ones only	214	54
Protect computer pass words	42	10.6
Obtain informed consent	74	18.7
Confidentiality after death	31	7.8
I don’t know	35	8.8
Read colleague’s notes (n = 316)		
Yes	230	72.8
No	86	27.2
Colleague’s notes fulfill standard (n = 230)		
Yes	100	43.5
No	130	56.5
Documents education or advice (n = 316)		
Always	116	36.7
Sometimes	109	34.5
Rarely	34	10.8
Never	57	18
Uses computerized documentation system(n = 316)		
Yes	54	17.1
No	262	82.9
Reports any medical error voluntarily(n = 316)		
Yes	225	71.2
No	91	28.8
Way of error recording(n = 225)		
No words like” error” or “mistake”	86	32.5
Facts only	132	49.8
I don’t know	47	17.7
Documents patient response to care (n = 316)		
Yes	187	59.2
No	129	40.8

#### Knowledge of respondents towards nursing documentation

A total of 10 multiple choice questions were used to measure the knowledge of respondents regarding nursing documentation and the mean score was 4.9 (SD ± 1.9). The minimum score was 1.5 and the maximum 9. The total mean score for knowledge questions was 4.9. Of all the respondents, 136 (43%) subjects scored above or equal to the mean value and the rest 180 (57%) of them scored below the mean. One hundred eighty (57%) of the respondents were found to have poor knowledge of documentation.

#### Attitude of respondents towards nursing documentation

Participants’ attitudes were assessed via a Likert scale, with item scores ranging from strongly agree (5) to strongly disagree (1) which had a potential score of 50. The total mean score for attitude was 42 (S.D ± 4.9) and scores greater or equal to the mean was categorized as favorable and unfavorable for scores below the mean. In this study the overall attitude score of the study participants showed that above half of respondents 176 (55.7%) had favorable attitude and the remaining 140 (44.3%) had unfavorable attitude.

#### Reason for poor nursing care documentation practice

Out of the 128 (40.5%) of respondents who do not document every care provided to a patient. Most 65 (41.9%) of them reported their reason to be lack of time followed by shortage of documenting sheets, inadequate staff, lack of motivation from supervisors and lack of obligation from employing institution by 38 (24.5%), 28 (18.1%), 17 (11%) and 7 (4.5%) respectively.

#### Factor associated with documentation practice of nursing care plan

Using binary logistic regression, crude odds ratio with 95% confidence interval was calculated to determine statistical significance and strength of association between each variable. Variables having a p value < 0.25 in the bivariate logistic analysis were entered into the multivariable logistic analysis and adjusted odds ratios were then calculated to investigate association with controlled confounding variables.

According to finding of this study, those nurses who are unfamiliar with operational standard of the nursing documentation were two times more likely to have poor nursing documentation practice than those who are familiar (AOR = 2.015, 95% CI 1.205, 3.370). Additionally, lacked time and those who lacked documentation sheets were two times [AOR = 2.205, 95% CI (1.101, 3.413)] and three times [AOR = 3.271, 95% CI (1.125, 23.704)] more likely to perform poor nursing documentation when compared to those with adequate time and adequate documenting sheets respectively (Table 3).

**Table 3:** Bivariate and multivariate logistic regression analysis for association between practice of nursing documentation with knowledge, attitude and organizational factors among nurses working in selected public hospitals of Tigray, Ethiopia, 2017

Variables	Practice level				Odds Ratio (95% CI)	
	Poor practice		Good practice		Crude (COR)	Adjusted(AOR)
	N	%	N	%		
Knowledge						
Poor	96	53.3	84	46.7	0.901 (0.577,1.407)	
Good	69	50.7	67	49.3	1	
Attitude						
Unfavorable	76	54.3	64	45.7	1	
Favorable	89	50.6	87	49.4	1.161 (0.744,1.811)	
Lack of sheets						
Yes	30	78.9	8	21.1	1.252 (1.111,0.569)^a^	3.271 (1.125, 23.704)^b^
No	135	48.6	143	51.4	1	1
Staff inadequacy						
Yes	18	64.3	10	35.7	0.252 (0.111,0.569)^a^	0.580 (0.236,1.425)
No	147	51	141	49	1	1
Time shortage						
Yes	53	81.5	12	18.5	1.182 (1.093,1.358)^a^	2.205 (1.101, 3.413)^b^
No	112	44.6	139	55.4	1	1
Lack of motivation						
Yes	14	82.4	3	17.6	0.219 (0.062,0.0776)^a^	0.263 (0.069,1.008)
No	151	50.5	148	49.5	1	1
Familiarity with hospital policy						
Unfamiliar	87	43.7	25	49	2.575 (1.600,4.143)^a^	2.015 (1.205,3.370)^b^
Familiar	26	51	14	21.2	1	1
Work setting						
Out-patient	91	58.7	64	41.3	1	1
In-patient	74	46	87	54	1.672 (1.071,2.609)^a^	1.591 (0.973,2.600)

^a^Factors associated with nursing documentation practice in bivariate analysis

^b^Factors associated with nursing documentation practice in multivariable analysis

### Discussion

This cross-sectional study aimed to investigate nursing documentation practice and associated factors among nurses in public hospitals of Tigray, Ethiopia.

The finding of this study showed that familiarity with operational standard of nursing documentation, lack of time and inadequacy of documenting sheets had a significant effect on nursing care documentation practice.

The result of this study shows that practice nursing care documentation was inadequate (47.8%) among nurses similar to Nigeria [24] where both the documentation practice and knowledge were found to be insufficient. This finding is higher from Indonesia 33.3% [23] and University of Gondar hospital (37.4%) [14]. This discrepancy might be due to difference in the study period since there might be information difference with time gap because the studies were done before 2 years and after technology had faster growth like smart care introduced in most hospitals of Ethiopia. The other reason could be nurses educational development variation across the countries [25]. Most (52.2%) of the study participants in this study revealed poor nursing documentation practice which coincides with a study done in Felege Hiwot referral hospital (87.5%) [19] where medication administration errors were due to nursing documentation error [19]. This finding is lower than a finding from South Africa 68.3% [22] and Nigeria 70% [15]. This might be due to insufficient knowledge as indicated in those studies favorability of the working environment and organizational structure.

Some barriers have been identified to hinder the nursing documentation practice in this study. Those nurses who are familiar with the availability operational standard of nursing documentation were two times more likely to document their care compared to the unfamiliar ones. Similarly, lack of time and scarcity of sheet were leading factors that negatively influence the nursing documentation practice in this study. Respondents who had lack of time were two times more likely to document (41.9%) similar to a study conducted in Nigeria (41.7%) [15] and England (47%).

Despite its non-significant association, knowledge has shown association with documentation practice in other studies. The knowledge level of participants was 43% in this study which contradicts with the finding from University of Gondar hospital (58.3%) [14], South Africa (74.9%) [22], Iraq (59%) [16] and Indonesia (82.7%) [23].These inconsistencies might be related to socio demographic variability of the study participants or difference in familiarity to the documentation guideline [14].

### Conclusions

Nursing care documentation practice was poor among nurses. Inadequacy of documenting sheets, lack of time and familiarity with operational standard of nursing documentation were factors associated with nursing care documentation practice. The following recommendation should forward to the healthcare facilities:

- Provide a training program to enhance the knowledge of nurses and to familiarize them with institutional policy regarding documentation and provide adequate documentation materials.

## Limitations

- Since this study is based on self-reported data, most of the variables might have been exposed for social desirability bias.

## Data Availability

The datasets used and/or analyzed during the current study are available from the corresponding author on reasonable request.

## Abbreviations

AOR: adjusted odd ratio
CI: confidence interval
FMOH: Federal Ministry of Health
IQR: inter quartile range
SPSS: Statistical Package for Social Sciences
